# Identification of Mild Cognitive Impairment with Traditional, Process, and Acoustic Metrics Derived from the Digital Assessment of Cognition

**DOI:** 10.1002/alz70856_103763

**Published:** 2025-12-26

**Authors:** Tanya Talkar, Daniel J Schulman, David J. Libon, Karl Thompson, Connor Higgins, Alvaro Pascual‐Leone, Sean Tobyne

**Affiliations:** ^1^ Linus Health, Boston, MA, USA; ^2^ Rowan University, Glassboro, NJ, USA; ^3^ Department of Psychology, Rowan University, Glassboro, NJ, USA; ^4^ Rowan‐Virtua School of Osteopathic Medicine, New Jersey Institute for Successful Aging, Stratford, NJ, USA; ^5^ Hebrew SeniorLife, Boston, MA, USA; ^6^ Harvard Medical School, Boston, MA, USA

## Abstract

**Background:**

Existing diagnostic criteria for mild cognitive impairment (MCI) or dementia due to Alzheimer's disease (AD) require documentation of cognitive impairment (CI). Traditional methods of assessment involve hours of neuropsychological testing. While this ensures a rigorous diagnosis, it raises a barrier for early detection of CI, which could delay a diagnosis and impact access to emerging treatments. In this exploratory study, we assess the utility of the remote Digital Assessment of Cognition (rDAC) and automatically extracted traditional, process, and acoustic metrics to discriminate between individuals with unimpaired cognition, MCI, and dementia.

**Methods:**

The rDAC was administered on an iPad to individuals identified as cognitively unimpaired (CU) (*n* = 24), MCI (*n* = 42), or dementia (*n* = 15) based on a three‐hour cognitive assessment battery and interview. The rDAC protocol is comprised of two 6‐word Philadelphia (repeatable) Verbal Learning Tests [P(r)VLT] immediate free recall trials, the semantic “animal” fluency test, three trials of the Backwards Digit Span Test (BDST), and the P(r)VLT delayed free recall and recognition tests. Derived metrics were used to train Random Forest models for four classification tasks: CU vs. MCI, CU vs. MCI+Dementia, CU+MCI vs. Dementia, and CU vs. Dementia.

**Results:**

We saw high performance in discrimination between CU and MCI with AUC=0.95, accuracy=0.87, sensitivity=0.88, and specificity=0.87. Detection of CI (CU vs. MCI+Dementia) had an AUC=0.98, accuracy=0.91, sensitivity=0.87, and specificity=0.95. The model for CU+MCI vs. Dementia performed similarly, with AUC=0.95, accuracy=0.88, sensitivity=0.84, and specificity=0.91. All performance metrics were 1.00 for discrimination between CU and Dementia. Important features included overall accuracy, speaking and articulation rate from P(r)VLT delayed recall, pause percentage from semantic fluency, jitter, and speech duration.

**Conclusions:**

The brief, iPad‐administered rDAC shows promise as a tool sensitive for flagging emergent CI, on par with neuropsychological testing. We believe the combination of traditional neuropsychological outcome measures, process metrics, and acoustic features will detect the subtle performance differences that distinguish CI in real world applications.

